# A New Discrete Analog of the Continuous Lindley Distribution, with Reliability Applications

**DOI:** 10.3390/e22060603

**Published:** 2020-05-28

**Authors:** Abdulhakim A. Al-Babtain, Abdul Hadi N. Ahmed, Ahmed Z. Afify

**Affiliations:** 1 Department of Statistics and Operations Research, King Saud University, Riyadh 11362, Saudi Arabia; hakim@ksu.edu.sa; 2 Department of Mathematical Statistics, Faculty of Graduate Studies for Statistical Research, Cairo University, Giza 12631, Egypt; dr.hadi@cu.edu.eg; 3 Department of Statistics, Mathematics and Insurance, Benha University, Benha 13511, Egypt

**Keywords:** discrete Lindley analog, extreme values, mean residual life, negative binomial distribution, COVID-19 data, estimation, characterization, reliability

## Abstract

In this paper, we propose and study a new probability mass function by creating a natural discrete analog to the continuous Lindley distribution as a mixture of geometric and negative binomial distributions. The new distribution has many interesting properties that make it superior to many other discrete distributions, particularly in analyzing over-dispersed count data. Several statistical properties of the introduced distribution have been established including moments and moment generating function, residual moments, characterization, entropy, estimation of the parameter by the maximum likelihood method. A bias reduction method is applied to the derived estimator; its existence and uniqueness are discussed. Applications of the goodness of fit of the proposed distribution have been examined and compared with other discrete distributions using three real data sets from biological sciences.

## 1. Introduction

Modeling of count data is found in many fields such as public health, medicine, epidemiology, applied science, sociology, and agriculture. Several distributions have been proposed for this count data, especially the count data with over-dispersion. However, it was found that the traditional discrete distributions (geometric, Poisson, etc.) have limited applicability as models for reliability, failure times, counts, etc. This is so, since many real count data show either over-dispersion, in which the variance is greater than the mean or under-dispersion, in which the variance is smaller than the mean. This has led to the development of some discrete distributions based on popular continuous models for reliability, failure times, etc.

On the other hand, it has been observed that many times in the real world the original variables may be continuous in nature but discrete by observation, e. g., modeling the number of motions of a pendulum before resting, the number of times devices are switched on/off, the number of days a patient stays in a hospital, and the number of weeks/months/years a kidney patient survives after treatment, the number of current fluctuations which an electrical item can withstand before its failure; among many other applications. Therefore, it is reasonable and convenient to model these situations by appropriate discrete distributions generated from the underlying continuous distributions preserving one or more important characteristics including probability density function (pdf), moment generating function (mgf), moments, hazard rate function (hrf), mean residual life function, etc. of the continuous distribution.

Interests in discrete failure data came relatively late in comparison to its continuous analogue. The subject matter has to some extent been neglected. It was only briefly mentioned by Barlow and Proschan [1]. For, earlier works on discrete lifetime distributions, see Salvia and Bollinger [2], Xekalaki [3], Padgett and Spurrier [4], and Ebrahimi [5].

In the last few decades, many papers have appeared in the statistical literature on the discretization of continuous distributions. In spite of all the available discrete models, there is still a great need to create more flexible discrete lifetime distributions to serve many areas like economics, social sciences, and biometrics, and reliability studies to suit various types of count data.

The most recent discrete distributions are those due to Krishna and Pundir [6], Jazi et al. [7], and Gómez-Déniz [8]. Krishna and Pundir [6] constructed discrete analogues of the continuous Burr and Pareto distributions. Jazi et al. [7] constructed a discrete analogue of the continuous inverse Weibull distribution. Gómez-Déniz [8] constructed a discrete analogue of the generalized exponential distribution due to Marshall and Olkin [9]. Being the most recent, these three distributions have not yet received any applications. All three distributions have at least two parameters each. All three distributions have moments expressed in terms of either infinite sums or non-standard special functions.

Several methods are available in the statistics literature to derive a discrete distribution from a continuous distribution. The most commonly used technique to generate discrete analogies from continuous ones is briefly described here. If the underlying continuous non-negative failure time X has the survival function (sf), S(x)=P[X≥x], and times are grouped into unit intervals, the corresponding probability mass function (pmf) is defined by

p(x)=S(x)−S(x+1), x=0, 1, 2, …

Several authors have used this discretization method of a continuous distribution to generate a corresponding discrete analog. Following this approach, the most recent discrete distributions are due to Stein and Dattero [10], Roy [11,12,13], Krishna and Pundir [6], Jazi et al. [7] and Gómez-Déniz [8].

Lindley [14] introduced the following continuous distribution function

$$F\left(x;\theta \right)=1-\frac{1+\theta +\theta x}{1+\theta }e^{-\theta x},\;x>0\;\mathrm{and}\;\theta >0$$

with the pdf

$$f\left(x;\theta \right)=\frac{\theta^{2}}{1+\theta }\left(1+x\right)e^{-\theta x}$$

to model various types of continuous lifetime data.

This distribution is derived as a mixture of exponential (θ) and gamma (2, θ) distributions.

It is worth noting that the pmfs of the two most recent discrete Lindley distributions due to Gómez-Déniz and Calderín-Ojeda [15] and that due to Bakouch et al. [16] are obtained by discretizing the continuous sf of the Lindley distribution and have a quite complex structure in terms of parameter estimation. In order to overcome problems in the estimation process of the parameter of Lindley distribution, we propose our new discrete Lindley distribution. To the best of our knowledge, this is the first article that uses the well-known fact that the geometric and the negative binomial distributions are the natural discrete analogs of the exponential and the gamma distributions, respectively (see, e.g., Nakagawa and Osaki [17] and Roy [13], among others).

To this end, we created a “natural” discrete analog of Lindley’s distribution, by mixing the geometric distribution and the negative binomial distribution.

We note that the model obtained is over-dispersed, which makes it suitable to be applied in the collective risk models and is competitive with the Poisson distribution to fit automobile claim frequency data.

**Definition** **1.** *Let X be a non-negative discrete random variable obtained as a finite mixture of geometric (θ) and negative binomial (2,θ) with mixing probabilities $\frac{\theta }{\theta +\beta }$ and $\frac{\beta }{\theta +\beta },$ respectively. The new TNDL distribution is specified by the pmf*

$$p\left(x;\theta ,\beta \right)=\frac{\theta^{2}}{\theta +\beta }{\left(1-\theta \right)}^{x}\left[1+\beta \left(1+x\right)\right],\;x=0,\;1,\;2,\ldots ,\;\beta >0\;\mathrm{and}\;\theta \in \left(0,1\right)$$

We note that the TNDL distribution includes the following discrete distributions as particular cases:

(i) The geometric distribution when β=0.
(ii) The discrete Lindley distribution of Bakouch et al. [16], when θ=1−p.

The corresponding cumulative distribution function (cdf), sf and hrf, denoted by r(x;θ,β), of the TNDL are given for x=0, 1, 2, …, β>0 and θ∈(0,1) by

$$F\left(x;\theta ,\beta \right)=P\left(X<x\right)=1-\frac{\theta \left(1+\beta \right)+\beta \left(1-\theta +\theta x\right)}{\theta +\beta }{\left(1-\theta \right)}^{x},$$

$$S\left(x;\theta ,\beta \right)=P\left(X\geq x\right)=\frac{\theta \left(1+\beta \right)+\beta \left(1-\theta +\theta x\right)}{\theta +\beta }{\left(1-\theta \right)}^{x}$$

and

$$r\left(x;\theta ,\beta \right)=\frac{\theta^{2}\left[1+\beta \left(1+x\right)\right]}{\theta \left(1+\beta \right)+\beta \left(1-\theta +\theta x\right)}$$

This new distribution can be considered as an alternative to the negative binomial, Poisson-inverse Gaussian, hyper-Poisson, and generalized Poisson distributions.

Although the two-parameter case has its merits, we decided to focus on the single parameter case, since the second parameter, β appears only in the mixing weights. Moreover, the one parameter case might be much more useful for practitioners and engineers.

Thus, our derivations have focused on of the single parameter natural discrete Lindley (NDL) distribution, i.e., TNDL when β=1. We note that the NDL distribution is the counterpart of the single parameter continuous Lindley distribution.

The rest of the paper is organized as follows. In Section 2, the discrete analog of the Lindley distribution is developed with some plots for its pmf and hrf. Some reliability characteristics of the NDL distribution along with some important theorems are established in Section 3. In Section 4, we develop explicit expressions for its moments. In Section 5, we introduce the entropy of the NDL model. A characterization of the NDL distribution in terms of a relationship between its mean residual life function and its hazard rate function is derived in Section 6. Section 7 provides the distribution of the maximum and the minimum in a random sample selected from the NDL distribution. We derive the asymptotic distribution of extreme order statistics in Section 8. In Section 9, the method of maximum likelihood and the method of moments are used to estimate the parameter θ. Section 9 applies a bias reduction method to the derived MLE estimator. Its existence and uniqueness are discussed along with simulation results to explore the behavior of the maximum likelihood estimator. Three real data sets are used to validate the use of NDL in fitting lifetime count data are presented in Section 10. Finally, conclusions are provided in Section 11.

## 2. The NDL Distribution

**Definition** **2.** *Let X be a non-negative random variable obtained as a finite mixture of geometric (θ) and negative binomial (2,θ) with mixing probabilities $\frac{\theta }{\theta +1}$ and $\frac{1}{\theta +1},$ respectively. The new distributions specified by the pmf*

(1) $$p\left(x;\theta \right)=\frac{\theta^{2}}{1+\theta }\left(2+x\right){\left(1-\theta \right)}^{x},\;x=0,\;1,\;2,\ldots \;\mathrm{and}\;\theta \in \left(0,1\right).$$

Lindley distribution may not be considered as a flexible model for analyzing different lifetimes and actuarial data. Therefore, to increase the flexibility for modeling purposes, we developed a single parameter NDL distribution. Furthermore, our new formulation provides a tractable model with attractive properties, which makes it suitable for applications not only in insurance settings but also in other fields where over-dispersions are observed. Some of these features include the uni-modality and over-dispersion. Many other properties and a recurrence formula for the probabilities of the new distribution are provided.

The corresponding sf of the NDL distribution is given by

$$S\left(x;\theta \right)=P\left(X\geq x\right)=\frac{1+\theta +\theta x}{1+\theta }{\left(1-\theta \right)}^{x},\;x=0,1,2,\ldots \;\mathrm{and}\;\theta \in \left(0,1\right)$$

Its hrf reduces to

$$r\left(x;\theta \right)=\frac{p\left(x\right)}{P\left(X\geq x\right)}=\frac{\theta^{2}\left(2+x\right)}{1+\theta +\theta x},\;\;\;\;x=0,1,2,\ldots \;\mathrm{and}\;\theta \in \left(0,1\right).$$

It is easy to see that $\underset{x\to \infty }{\lim}r\left(x;\theta \right)=\theta .$ Hence, the parameter θ can be interpreted as a strict upperbound on the failure rate function, an important characteristic for lifetime models, corresponding to Equation (1). Not many discrete distributions have their parameters directly interpretable in terms of their failure rate functions. One exception is the geometric distribution but in this case the failure rate function is a constant. We shall also see later that the NDL distribution always allows for increasing failure rates. It does not allow for a constant or decreasing failure rate. The geometric, discrete Weibull and discrete gamma distributions do allow for constant or decreasing failure rates. These are very unrealistic features because there are hardly any real-life systems that have constant or decreasing failure rates. So, the NDL distribution is more useful than the geometric distribution for modeling the number of rare events. Furthermore, when θ is closed to zero, then the NDL distribution can have different shapes than the pmf of a geometric distribution. This situation made our distribution have a thinner right tail than a distribution, which is compounded with exponential distribution. Hence, the new NDL distribution can be useful for modeling lifetime data such as a time interval between successive earthquakes, the time period of bacteria spreading, and the recovery period of a certain disease.

**Figure 1:**
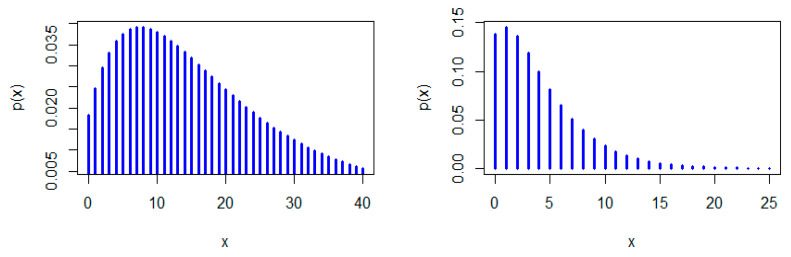
Probability mass function (pmf) plots of the natural discrete Lindley (NDL) distribution: θ=0.1 (**left panel**) and θ=0.3 (**right panel**).

**Figure 2:**
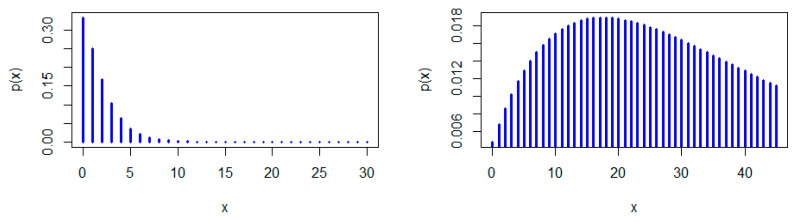
pmf plots of the NDL distribution: θ=0.5 (**left panel**) and θ=0.05 (**right panel**).

**Figure 3:**
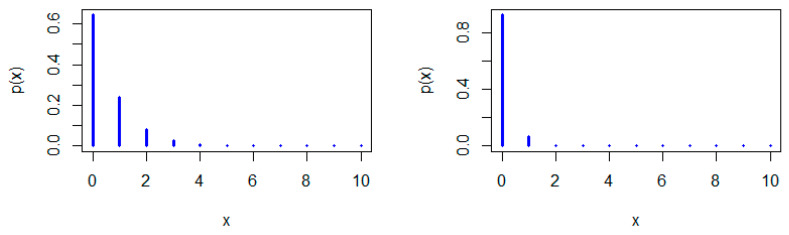
pmf plots of the NDL distribution: θ=0.75 (**left panel**) and θ=0.95 (**right panel**).

**Figure 4:**
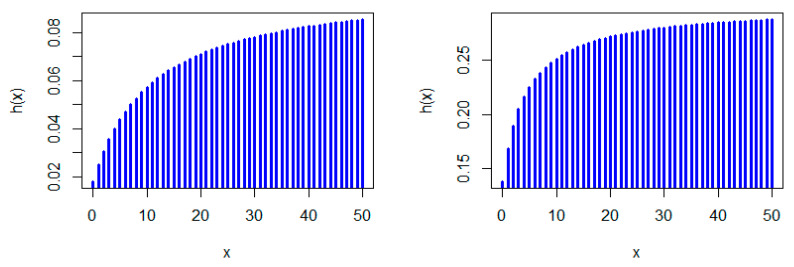
Hazard rate function (hrf) plots of the NDL distribution: θ=0.1 (**left panel**) and θ=0.3 (**right panel**).

**Figure 5:**
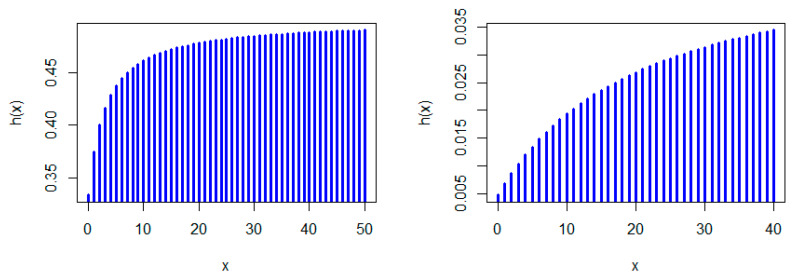
hrf plots of the NDL distribution: θ=0.5 (**left panel**) and θ=0.05 (**right panel**).

Figure 1, Figure 2 and Figure 3 show some possible shapes for the pmf of the NDL distribution. One can note that the NDL distribution is always uni-modal for any value of θ (see also Theorem 1). Figure 4 and Figure 5 indicate that the hrf of the NDL distribution is always increasing in θ (see also Theorem 1).

## 3. Reliability Properties of NDL Distribution

### 3.1. Log-Concavity

**Definition** **3.** *A discrete random variable X with pmf p(x) is said to be increasing failure rate (IFR) if p(x) is log-concave, i.e., if $p\left(x\right)p\left(x+2\right)\leq p{\left(x+1\right)}^{2},\;x=0,1,2,\ldots$ (see, e.g., Keilson and Gerber [18]).*

**Theorem** **1.** *The pmf of the NDL distribution in (1) is log-concave for all choices of θ∈(0,1).*

**Proof.**  The condition in Definition (3) is easily verified from (1).  □

Generally, it is well-known that a log-concave pmf is strongly uni-modal (see, e.g., Nekoukhou et al. [19]) and accordingly have the discrete IFR property, (see, e.g., Barlow and Proschan [1]. It follows from Theorem 1 that the NDL distribution is uni-modal and has the discrete IFR property (see Figure 1, Figure 2, Figure 3, Figure 4 and Figure 5). Thus, we have the following corollary.

**Corollary** **1.** *If the random variable X~NDL(θ) then the mode of X is located at w, where w is a positive integer satisfies $\frac{1-3\theta }{\theta }\leq w\leq \frac{1-2\theta }{\theta }.$ This implies that p(x+1)≥p(x) ∀x≤w and p(x+2)≤p(x+1) ∀x≥w (see, e.g., Kielson and Gerber [18], Nekoukhou et al. [19], and Abouammoh and Mashhour [20]). Hence, the NDL distribution has the following chain of implications IFR⇒IFRA⇒NBU⇒NBUE⇒DMRL (see, Kemp [21]), where IFRA refers to increasing failure rate average, NBU refers to new better than used, NBUE refers to new better than used in expectation and DMRL refers to decreasing mean residual lifetime.*

**Definition** **4.** *A discrete life distribution $P=\left\{p_{k}=P\left(X=k\right)\right\},\;k\in N,$ where N is the set of all non-negative integers. With $A_{k}=P\left(X\leq k\right),$ we define the discrete reversed failure rate (DRFR) as follows*

$$r_{k}^{*}=\frac{p_{k}}{A_{k}},\;k\in N.$$

**Definition** **5.** *(Al-Zahrani and Al-Sobhi [22]): A discrete life distribution $P=\left\{p_{k}=P\left(X=k\right)\right\},\;k\in N,$ where N is the set of all non-negative integers is said to be discrete increasing (decreasing) reversed failure rate DIRFR (DDRFR) if $r_{k}^{*},\;k\in N$ is increasing (decreasing).*

**Proposition** **1.** *Let $p_{k}$ the sequence defined by the NDL distribution, then NDL distribution has the DIRFR property.*

**Proof.**  It is easy to prove that $r_{k}^{*}$ is increasing in k.  □

The reversed hrf of X is

$$r^{*}\left(x;\theta \right)=\frac{p\left(x;\theta \right)}{1-S\left(x;\theta \right)}=\frac{\left(1+\theta +\theta x\right){\left(1-\theta \right)}^{x}}{\left[\left(1+\theta \right)-\left(1+\theta +\theta x\right){\left(1-\theta \right)}^{x}\right]}.$$

**Remark** **1.** *The following is a simple recursion formula for* p(x+1) *in terms of* p(x) *of the NDL for* x=0, 1, 2, … *, where*

$$p\left(x+1\right)=\frac{3+x}{2+x}\left(1-\theta \right)p\left(x\right),$$

*where* $p\left(0\right)=2\theta^{2}/\left(1+\theta \right).$

**Remark** **2.**

*(i)*  r(0)=p(0).
*(ii)*  r(x) *is an increasing function in x*and*θ.*
*(iii)*  r(x)≥r(0) ∀ x∈N *and hence the NDL distribution has the new better thanused in failure rate (NBUFR) property (see, Abouammoh and Ahmed [23]).*

**Remark** **3.** *Following Salvia and Bollinger [2], the following bounds hold for the sf, the mean, and the MRL function. For any* k=1, 2, …

$$S\left(k\right)\leq 1-r\left(1\right)=\frac{\left(3\theta +1\right)\left(1-\theta \right)}{1+2\theta },$$

$$\mu_{1}^{\prime }\leq \frac{1-r\left(1\right)}{r\left(1\right)}=\frac{\left(3\theta +1\right)\left(1-\theta \right)}{3\theta^{2}}$$

*and*

$$1-\frac{\theta^{2}\left(2+k\right)}{1+\theta +\theta k}\leq \mu_{1}^{\prime }\leq \frac{\left(3\theta +1\right)\left(1-\theta \right)}{3\theta^{2}}.$$

### 3.2. Stochastic Interpretations of the Parameter Theta

Stochastic orders are important measures to judge comparative behaviors of random variables. Shaked and Shanthikumar [24] showed that many stochastic orders exist and have various applications.

**Definition** **6.** *Let X and Y be two random variables with cumulative distribution functions $F_{X}\left(.\right)$ and $F_{Y}\left(.\right),$ respectively.*

*(i)*  *Stochastic order ($X\leq_{st}Y$): if $F_{X}\left(x\right)\geq F_{Y}\left(x\right)$ for all x.*
*(ii)*  *Hazard rate order ($X\leq_{hr}Y$): if $r_{X}\left(x\right)\geq r_{Y}\left(x\right)$ for all x.*
*(iii)*  *Reversed hazard rate order ($X\leq_{rh}Y$): if $r_{X}^{*}\left(x\right)\leq r_{Y}^{*}\left(x\right)$ for all x.*
*(iv)*  *Mean residual life order ($X\leq_{mrl}Y$): if $m_{X}\left(x\right)\leq m_{Y}\left(x\right)$ for all x.*
*(v)*  *Likelihood ratio order ($X\leq_{lr}Y$): if $p_{X}\left(x\right)/p_{Y}\left(x\right)$ is non-decreasing in x.*

*The following chains of implication (see, e.g., Shaked and Shantihkumar [24]) hold.*

$$X\leq_{lr}Y\Rightarrow \begin{array}{c} X\leq_{hr}Y\\ \begin{array}{c} ⇓\\ X\leq_{st}Y \end{array} \end{array}\Rightarrow X\leq_{mrl}Y\;\mathrm{and}\;X\leq_{lr}Y\Rightarrow X\leq_{rh}Y$$

**Theorem** **2.** *Let $\sim NDL\left(\theta_{1}\right)$ and $Y\sim NDL\left(\theta_{2}\right).$ Then, $X\leq_{lr}Y$ for all $\theta_{1}>\theta_{2}.$*

**Proof.**  We have

$$L\left(x\right)=\frac{p_{X}\left(x\right)}{p_{Y}\left(x\right)}=\frac{\theta_{1}^{2}\left(1+\theta_{2}\right){\left(1-\theta_{1}\right)}^{x}}{\theta_{2}^{2}\left(1+\theta_{1}\right){\left(1-\theta_{2}\right)}^{x}}.$$

Clearly, one can see that $L\left(x+1\right)\leq L\left(x\right)\;\forall \;\theta_{1}>\theta_{2}.$

Theorem 2 shows that the NDL distribution is ordered according to the strongest stochastic order (v).  □

**Corollary** **2.** *Based on the chain of stochastic orders in the definition (4),* $X\leq_{hr}Y,X\leq_{rh}Y,X\leq_{mrl}Y$ *and* $X\leq_{st}Y.$

**Definition** **7.** *The discrete random variable X is said to be smaller than Y in weak likelihood ratio ordering (denoted by $X\leq_{wlr}Y$) if $\frac{p_{X}\left(x+1\right)}{p_{Y}\left(x+1\right)}\leq \frac{p_{X}\left(0\right)}{p_{Y}\left(0\right)}\;\forall \;x\geq 0$ (see, Khider et al. [25]).*

**Theorem** **3.** *Let $\sim NDL\left(\theta_{1}\right)$ and $Y\sim NDL\left(\theta_{2}\right)$. Then, X is said to be smaller than Y in weak likelihood ratio ordering, denoted by $X\leq_{wlr}Y,$ for all $\theta_{1}<\theta_{2}.$*

**Proof.**  According to Definition 2.5 of Khider et al. [25], we can prove that

$$\frac{p_{X}\left(x+1\right)}{p_{Y}\left(x+1\right)}\leq \frac{p_{X}\left(0\right)}{p_{Y}\left(0\right)}.$$

Then, we obtain

$$\frac{\theta_{1}^{2}\left(1+\theta_{2}\right)\left[{\left(1-\theta_{1}\right)}^{x+1}-{\left(1-\theta_{2}\right)}^{x+1}\right]}{\theta_{2}^{2}\left(1+\theta_{1}\right){\left(1-\theta_{2}\right)}^{x+1}}\leq 0,\;\forall \;\theta_{1}<\theta_{2}.$$

Hence $X\leq_{wlr}Y.$

The mean residual life (MRL) function of the NDL distribution is defined by

$$m\left(x\right)=E\left(X-x|X\geq x\right)=\frac{1-\theta }{\theta^{2}}r\left(x\right)+\frac{\left(1-\theta \right)\left(2-\theta \right)}{\theta \left(1+\theta +\theta x\right)},$$

where r(x) is the hrf of the NDL distribution.  □

**Theorem** **4.** *(Stochastic comparisons of partial random sums): Let* $\left\{X_{i},\;i=1,2,\ldots \right\}$ *be a sequence of NDL random variables, and* M *and* N *be two NDL random variables and independent of* ${X_{i}}^{\prime }$ *s. Then*

$$\overset{M}{\underset{i=0}{\sum }}X_{i}\begin{array}{c} \leq \\ mrl \end{array}\overset{N}{\underset{i=0}{\sum }}Y_{i}.$$

**Proof.**  Follows directly from Corrolary and Theorem 1.B.4 in Shaked and Shanthikumar [26].  □

## 4. Moments

The first four raw moments of the NDL distribution are, respectively, given by

(3) $$\mu_{1}^{\prime }=E\left(X\right)=\frac{\left(1-\theta \right)\left(2+\theta \right)}{\theta \left(1+\theta \right)},$$

$$\mu_{2}^{\prime }=\frac{\theta^{3}+\theta^{2}-8\theta +6}{\theta^{2}\left(1+\theta \right)},$$

$$\mu_{3}^{\prime }=\frac{\left(1-\theta \right)\left(\theta^{3}+2\theta^{2}-24\theta +24\right)}{\theta^{3}\left(1+\theta \right)}$$

and

$$\mu_{4}^{\prime }=\frac{\left(1-\theta \right)\left(-\theta^{4}-2\theta^{3}+78\theta^{2}-192\theta +120\right)}{\theta^{4}\left(1+\theta \right)}.$$

The corresponding variance and index of dispersion (ID) are

$$\mathrm{Variance}\left(X\right)=\frac{\left(1-\theta \right)\left(4\theta +2\right)}{\theta^{2}{\left(1+\theta \right)}^{2}}$$

and

$$\mathrm{ID}\left(X\right)=\frac{\mathrm{Variance}\left(X\right)}{E\left(X\right)}=\frac{2\left(2\theta +1\right)}{\theta \left(1+\theta \right)\left(2+\theta \right)}.$$

The ID indicates whether a certain distribution is suitable for under or over-dispersed data sets and has applications in ecology for measuring clustering (see, e.g., Johnson [27]). If the ID≥1, the distribution is over-dispersed. It is observed that the distribution shows over-dispersion for all values of θ. We note that the ID decreases monotonically in θ. It converges to 1 as θ→1, while it tends to infinity when θ→0. So, the NDL distribution should only be used in the count data analysis with over-dispersion. In Table 1 a set of numerical values for different values of θ are shown for practical uses. It is noted from Table 1 that the mean, variance, and ID are all decreasing functions of θ.

The moment generating function is

$$M_{X}\left(t\right)=E\left(e^{tX}\right)=\frac{\theta^{2}\left(2-\bar{\theta }e^{t}\right)}{\left(1+\theta \right){\left(1-\bar{\theta }e^{t}\right)}^{2}},t<\log\left(1-\theta \right)\;\mathrm{and}\;\theta \in \left(0,1\right).$$

The probability generating function is

$$\Psi_{X}\left(s\right)=E\left(s^{X}\right)=\frac{\theta^{2}\left(2-\bar{\theta }s\right)}{\left(1+\theta \right){\left(1-\bar{\theta }s\right)}^{2}},\left|t\right|<1/\left(1-\theta \right).$$

The kth descending factorial moment of X is given (for k=0,1,2,…) by

$$\mu_{\left(k\right)}^{\prime }=\frac{\left(k+\theta +1\right){\left(1-\theta \right)}^{k}k!}{\left(1+\theta \right)\theta^{k}},$$

where $\mu_{\left(k\right)}^{\prime }=E\left[X\left(X-1\right)\ldots \left(X-k+1\right)\right].$ Clearly, for k=0, we obtain $\mu_{\left(0\right)}^{\prime }=1$ and the mean of X in (2) follows as $\mu_{\left(1\right)}^{\prime }=E\left(X\right).$

The kth ascending factorial moment of X is given (for k=0, 1, 2,…) by

$$\mu_{\left[k\right]}^{\prime }=\frac{\left[k{\left(1-\theta \right)}^{2}+1+\theta -2\theta^{2}\right]k!}{\left(1+\theta \right)\theta^{k}},$$

where $\mu_{\left[k\right]}^{\prime }=E\left[X\left(X+1\right)\ldots \left(X+k-1\right)\right].$ Clearly, for k=0, we obtain $\mu_{\left[0\right]}^{\prime }=\frac{1+\theta -2\theta^{2}}{1+\theta }$ and the mean of X in (2) follows as $\mu_{\left[1\right]}^{\prime }=E\left(X\right).$

## 5. Entropy

The entropy is a measure of uncertainty of a random variable and it can be defined for a discrete random variable with pmf, p(x), by the formula (Gray [28]).

$$H\left(X\right)=-\underset{x}{\sum }p\left(x\right)\log p\left(x\right).$$

The entropy of the NDL distribution can be calculated by Mathematica software© var.9. The following formula is obtained by Mathematica as follows.

$$H\left(X\right)=-\log\left(\frac{\theta^{2}}{1+\theta }\right)+\frac{\theta^{2}}{1+\theta }{\mathrm{LerchPhi}}^{\left(0,1,0\right)}\left[1-\theta ,-1,2\right]+\frac{\left(\theta^{2}+\theta -2\right)}{\theta \left(1+\theta \right)},$$

where ${\mathrm{LerchPhi}}^{\left(0,1,0\right)}\left[z,a,s\right]$ gives the Lerch transcendent $\Phi \left(z,a,s\right)=\sum_{k=}^{\infty }\frac{z^{k}}{{\left(k+s\right)}^{a}}.$

**Figure 6:**
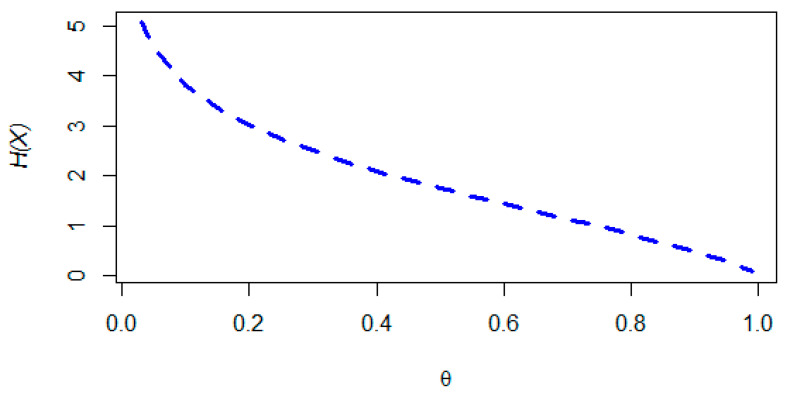
Entropy of X, H(X), versus θ.

Table 2 presents some numerical values of the entropy of an NDL (θ) for different choices of θ. Using the Mathematica software^©^ var.9, Figure 6 relates the entropy, H(X), to the values of parameter θ. One may note that H(X) is monotonically decreasing in θ∈(0,1) with its limit tending to zero as θ tends to 1.

## 6. Characterization

In this section, we characterize the NDL distribution in terms of a relationship between its mean residual life function and its hazard rate function. This is given in the following theorem.

**Theorem** **5.** *Let X be a non-negative discrete random variable with pmf P(X=x) and x=0,1,2,…, it then will follow the NDL distribution with parameter θ if*

(4) $$m\left(x\right)=E\left(X-x|X\geq x\right)=\frac{1-\theta }{\theta^{2}}r\left(x\right)+\frac{\left(1-\theta \right)\left(2-\theta \right)}{\theta \left(1+\theta +\theta x\right)},\;\forall x,$$

*where r(x) is the hrf of the NDL distribution.*

**Proof.**   

**Necessity.** The MRL function is defined as (see, Kemp [21])

$$m\left(x\right)=\frac{\sum_{k=x+1}^{\infty }S\left(k\right)}{S\left(x\right)}.$$

This implies that

$$m\left(x\right)=\frac{\left(1+\theta \right)\sum_{k=x+1}^{\infty }{\left(1-\theta \right)}^{k}+\theta \sum_{k=x+1}^{\infty }k{\left(1-\theta \right)}^{k}}{\left(1+\theta \right)S\left(x\right)}$$

$$m\left(x\right)=\frac{\theta {\left(1-\theta \right)}^{x+1}\left(2+\theta +\theta x\right)}{\theta^{2}\left(1+\theta \right)S\left(x\right)}.$$

After some simplification, one has

$$m\left(x\right)=\frac{\left(1-\theta \right)r\left(x\right)}{\theta^{2}}+\frac{\left(1-\theta \right)\left(2-\theta \right)}{\theta \left(1+\theta +\theta x\right)}.$$

 □

**Sufficiency:** Suppose that Equation (3) holds, then we can rewrite it as

$$\overset{\infty }{\underset{k=x+1}{\sum }}S\left(k\right)=\frac{\left(1-\theta \right)p\left(x\right)}{\theta }+\frac{\left(1-\theta \right)\left(2-\theta \right)}{1+\theta }.$$

Or

$$\overset{\infty }{\underset{k=x+1}{\sum }}S\left(k\right)=\frac{p\left(x+1\right)}{\theta }+\frac{2{\left(1-\theta \right)}^{2}{\left(1-\theta \right)}^{x}}{1+\theta }.$$

Comparing the last two equations, we obtain

$$p\left(x+1\right)-\left(1-\theta \right)p\left(x\right)=\frac{\theta^{2}{\left(1-\theta \right)}^{x+1}\left(2+x\right)}{\left(1+\theta \right)\left(2+x\right)}$$

(2+x)p(x+1)=(3+x)(1−θ)p(x)

which gives

$$p\left(0\right)=\frac{2\theta^{2}}{1+\theta }\;\mathrm{and}\;p\left(x\right)=p\left(0\right)\frac{\left(2+x\right)}{2}{\left(1-\theta \right)}^{x}$$

**Remark** **4.**

*(i)*  m(0)=μ.
*(ii)*  $m\left(x\right)=\frac{\left(1-\theta \right)\left(2+\theta +\theta x\right)}{\theta \left(1+\theta +\theta x\right)}$ *is a decreasing function in x and in θ.*

## 7. Distribution of the Maximum and the Minimum in a Random Sample from the NDL Distribution

Maximum and minimum of random variable arise in reliability. Let $X_{i},\;i=1,2,\ldots ,n,$ be iid random variables from the NDL distribution with parameter θ. Then, the sf of the minimum, $\mathrm{Min}\left(X_{1},\;X_{2},\;\ldots ,X_{n}\right)$, is given by

$${\bar{F}}_{\mathrm{Min}}\left(x\right)={\left(\frac{1+\theta +\theta x}{1+\theta }\right)}^{n}{\left(1-\theta \right)}^{nx}.$$

The cdf of the maximum, $\mathrm{Max}\left(X_{1},\;X_{2},\ldots ,X_{n}\right)$, is given by

$$F_{\mathrm{Max}}\left(x\right)={\left(1-\frac{1+\theta +\theta x}{1+\theta }\right)}^{n}{\left(1-\theta \right)}^{nx}.$$

## 8. Asymptotic Distribution of Extreme Order Statistics

Sometimes it is of interest to consider the asymptotic distributions of the extreme order statistics, that is, $X_{1:n}$ and $X_{n:n}$. One can see that

$$\underset{t\to \infty }{\lim}\frac{1-F\left(t+x/\theta \right)}{1-F\left(t\right)}=\underset{t\to \infty }{\lim}\frac{\left(1+\theta +x+\theta t\right){\left(1-\theta \right)}^{x/\theta }}{\left(1+\theta +\theta t\right)}={\left(1-\theta \right)}^{x/\theta }.$$

It can also be shown that

$$\underset{t\to 0}{\lim}\frac{F\left(tx\right)}{F\left(t\right)}=\underset{t\to 0}{\lim}\frac{1+\theta -\left(1+\theta +\theta tx\right){\left(1-\theta \right)}^{tx}}{1+\theta -\left(1+\theta +\theta t\right){\left(1-\theta \right)}^{t}}=x.$$

Hence, it follows from Theorem 1.6.2 in Leadbetter et al. [29] that there must be norming constants $a_{n}>0,\;b_{n},\;c_{n}>0$ and $d_{n}$ such that

$$\mathrm{Pr}\left\{a_{n}\left(X_{n:n}-b_{n}\right)\leq t\right\}\to \exp\left[-{\left(1-\theta \right)}^{x/\theta }\right]$$

and

$$\mathrm{Pr}\left\{c_{n}\left(X_{1:n}-d_{n}\right)\leq t\right\}\to 1-{\left(1-\theta \right)}^{\frac{x}{\theta }}.$$

as n→∞.

## 9. Estimation and Simulation

In this section, we estimate the parameter θ of the NDL distribution, using both maximum likelihood estimator (MLE) and moment estimator (ME). We show that they result in the same estimator in closed form. We note that the maximum likelihood method is often adopted to estimate the unknown parameters of a statistical model because the maximum likelihood estimators (MLEs) have many appealing properties; for example, they are asymptotically unbiased, consistent, and asymptotically normally distributed, etc.

Let $x_{1},\ldots ,x_{n}$ be a random sample of size n from the NDL distribution, then the log-likelihood function is given by

$$\ell \left(\theta |x\right)\propto 2n\log\left(\theta \right)-n\log\left(1+\theta \right)+n\bar{x}\log\left(1-\theta \right).$$

The MLE of θ follows by solving $\frac{d}{d\theta }\ell \left(\theta |x\right)=0$, that is

$$\frac{d}{d\theta }\ell \left(\theta |x\right)=\frac{2n}{\theta }-\frac{n}{1+\theta }-\frac{n\bar{x}}{1-\theta }=0.$$

After some algebra, the MLE of θ, is given by the following compact formula

$$\hat{\theta }=\frac{1}{2}\sqrt{1+8/\left(1+\bar{x}\right)}-\frac{1}{2}.$$

In the recent work of Balabdaoui et al. [30], the discrete MLE under the constraint of log-concavity was studied. As opposed to the continuous setting, existence of the uni-modal MLE when the data are discrete is guaranteed. On the other hand, uniqueness is not always true, but this problem is rather marginal, as a rule for selecting from among the finite options is immediate, making our estimator fully automatic and easy to compute.

Following the bias reduction technique presented in Reath et al. [31], one can reach the following formula for the bias-correction (BI-C):

(5) $$\mathrm{BI-C}\left(\hat{\theta }\right)=-\frac{1}{n^{2}}\left[\frac{1}{\theta^{3}}+\frac{1}{{\left(1+\theta \right)}^{3}}+\frac{\left(1-\theta \right)\left(2+\theta \right)}{\theta \left(1+\theta \right){\left(1-\theta \right)}^{3}}\right].$$

Next, we present the results of a conducted simulation study to explore the behavior of the maximum likelihood estimator with and without bias reduction for different combinations of the parameter θ and samples sizes, usind the measures average estimate (AVE), average mean square error (MSE), average bias (ABI), BI-C and average mean relative error (MRE).

The MSE, ABI, BI-C, and MRE are defined (for θ) by MSE $=\frac{1}{N}\sum_{i=1}^{N}{\left(\hat{\theta }-\theta \right)}^{2}$, ABI $=\frac{1}{N}\sum_{i=1}^{N}\left|\hat{\theta }-\theta \right|$, and MRE $=\frac{1}{N}\sum_{i=1}^{N}|\hat{\theta }-\theta |/\theta$.

**Figure 7:**
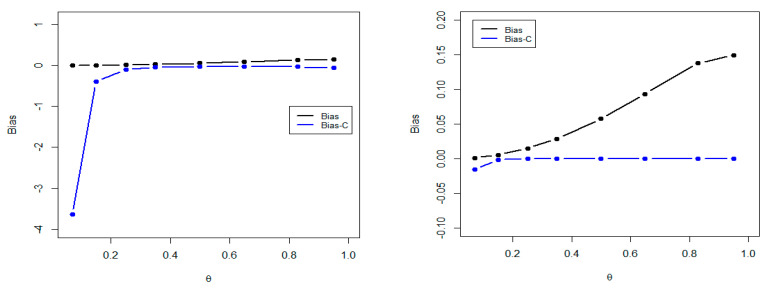
Comparison of the bias and bias corrected for n=30 (**left panel**) and n=300 (**right panel**).

We generated 5000 random samples of sizes n=(20, 30, 50, 100, 150, 300) from NDL (θ) distribution using the inversion method. The numerical results are obtaind using the R software, for values θ=(0.07, 0.15, 0.25, 0.35, 0.50, 0.65, 0.83, 0.95). The AVE, MSE, ABI, BI-C, and MRE for θ are reported in Table 3. It is clear, from Table 3, that the estimates of θ are very close to the true values for all values of θ Furthermore, for illustration Figure 7 presents comparisons between the estimators with and without bias corrections for n=20 and n=300.

It is clear, from the above figure, that the difference in bias is noticed for values of θ less than 0.3 and almost disappears for values equal to or more than 0.3 when the sample size is 20, while the difference in bias is quite obvious when θ exceeds 0.3 and the sample size is 300.

## 10. Applications to Count Data

In this section, we use three real data sets to illustrate the importance and superiority of the NDL distribution over the existing models, namely discrete Lindley (DL) [16], discrete Burr (DB), geometric (Gc), discrete Pareto (DP) [6], and discrete Burr-Hatke (El-Morshedy et al. [32]) distributions. The first dataset consists of remission times in weeks for 20 leukemia patients randomly assigned to a certain treatment (Lawless [33]).

The second data set consists of 123 observations, and it refers to numbers of fires, only fires in forest districts are considered, in Greece for the period from 1 July 1998 to 31 August of the same year (Karlis and Xekalaki [34]). Both data sets have been analyzed and reported by [16].

The third data set represents the numbers of daily deaths in Egypt due to COVID-19 infections from 8 March to 30 April, 2020, and contains 47 observations which are reported on worldometer website through https://www.worldometers.info/coronavirus/country/egypt/. The data are: 1, 1, 2, 2, 1, 1, 2, 4, 5, 1, 1, 3, 6, 6, 4, 1, 5, 6, 6, 8, 5, 7, 7, 9, 9, 15, 17, 11, 13, 5, 14, 5, 13, 9, 19, 15, 11, 14, 12, 11, 7, 13, 10, 20, 22, 21, 12. Some summary statistics for the three datasets are shown in Table 4.

The maximum likelihood estimates, their standard errors, and Kolmogorov–Smirnov (KS) statistics with their associated *p*-values are reported in Table 5. It is shown, from Table 4, that the new NDL distribution provides better fits for the three data sets over the DL, Gc, DB, DP, and DBH models.

**Figure 8:**
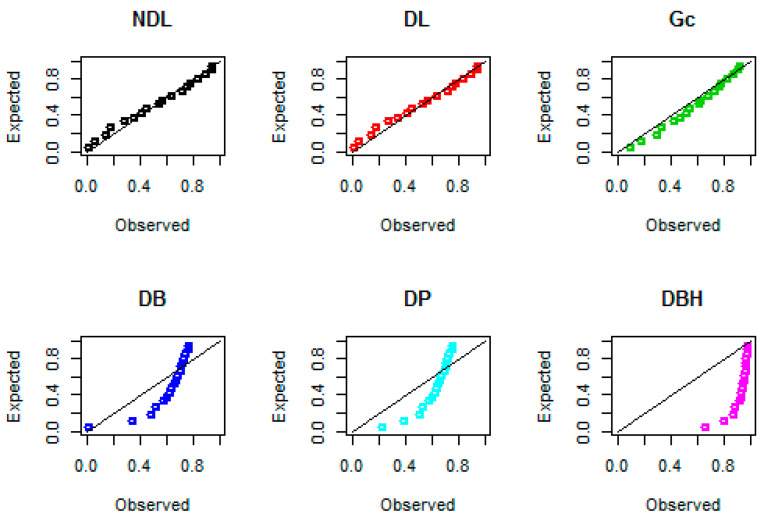
Probability–probability (PP) plots for data set I.

**Figure 9:**
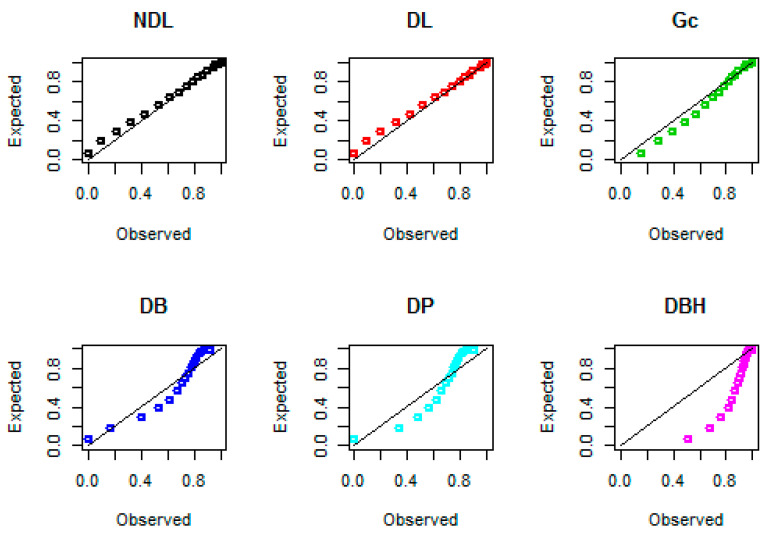
PP plots for data set II.

**Figure 10:**
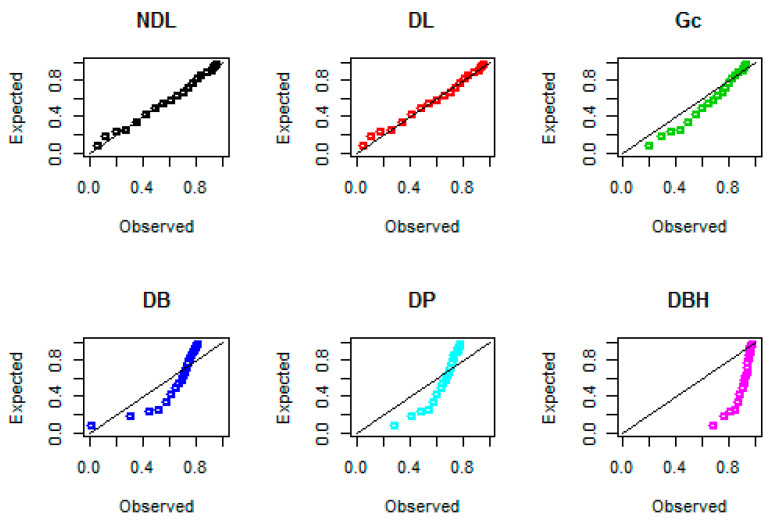
PP plots for data set III.

Probability–probability (PP) plots for the three data sets are shown in Figure 8, Figure 9 and Figure 10, respectively. These plots support the results in Table 5, that the NDL provides a closer fit for the emission times, numbers of fires, and Coronavirus data compared to DL, Gc, DB, DP, and DBH distributions.

## 11. Conclusions

In this paper, we propose and study a new natural discrete analog of the continuous Lindley distribution as a mixture of geometric and negative binomial distributions. The new distribution is called natural discrete Lindley (NDL) distribution and it has many interesting properties that make it superior to many other discrete distributions, particularly in analyzing over-dispersed count data. The moments of the NDL distribution and many reliability properties are derived in closed forms. A characterization of the NDL distribution relating its mean residual life function and its hazard rate function is derived and used to characterize the NDL distribution. We also provide the distribution of the maximum and the minimum in a random sample selected from the NDL distribution.

The maximum likelihood and moment estimators the parameter *θ* are derived and the bias reduction technique is applied. Simulation results to explore the MLE behavior and to compare between the bias and bias corrected are conducted. Three real data sets are used to validate the use of NDL in fitting lifetime count data.

It is worth mentioning that the research in this paper can be extended in many ways. For example, two or three-parameter NDL could be considered together with extensive bias reduction techniques.

Transmuted and/or exponentiated versions may be established, several properties of order statistics from the distribution could be explored and their relations to well-known stochastic orders, and a bivariate discrete NDL may also be studied.

## Figures and Tables

**Table 1:** Numerical values of mean, variance, and index of dispersion (ID).

θ	Mean	Variance	ID
0.01	197.0198	19,798.06	100.4877
0.05	37.0952	758.2766	20.4413
0.09	19.3873	223.1595	11.5105
0.10	17.1818	178.5124	10.3896
0.20	7.3333	38.8888	5.3030
0.30	4.1282	14.7271	3.5674
0.40	2.5714	6.8877	2.6785
0.50	1.6666	3.5555	2.1333
0.60	1.0833	1.9097	1.7628
0.70	0.6806	1.0168	1.4939
0.80	0.3888	0.5015	1.2896
0.90	0.1695	0.1915	1.1292
0.95	0.0796	0.0845	1.0613
0.99	0.0151	0.0153	1.0117

**Table 2:** Entropy of X versus θ.

θ	H(X)	θ	H(X)	θ	H(X)
0.03	5.06555	0.35	2.29581	0.70	1.13240
0.05	4.54033	0.40	2.10362	0.75	0.97926
0.10	3.80629	0.45	1.92492	0.80	0.82278
0.15	3.35507	0.50	1.75615	0.85	0.65954
0.20	3.01817	0.55	1.59462	0.90	0.48397
0.25	2.74307	0.60	1.43811	0.95	0.28415
0.30	2.50644	0.65	1.28467	0.99	0.07885

**Table 3:** Simulation results for the NDL(*θ*).

n		20	30	50	100	150	300
AVE	θ=0.07	0.06971	0.06945	0.06904	0.06901	0.06899	0.06886
MSE		0.00008	0.00004	0.00002	0.00001	0.00001	0.00001
ABI		0.00029	0.00055	0.00096	0.00099	0.00101	0.00114
BI-C		3.63772	−1.27307	−0.31543	−0.13907	−0.03452	−0.01538
MRE		0.10028	0.07619	0.05407	0.04439	0.03307	0.02872
AVE	θ=0.15	0.14664	0.14546	0.14506	0.14500	0.14477	0.14472
MSE		0.00031	0.00019	0.00011	0.00008	0.00006	0.00005
ABI		0.00336	0.00454	0.00494	0.00500	0.00523	0.00528
BI-C		−0.40219	−0.14380	−0.03537	−0.01562	−0.00390	−0.00173
MRE		0.09436	0.07546	0.05587	0.04870	0.04126	0.03831
AVE	θ=0.25	0.23795	0.23627	0.23573	0.23565	0.23532	0.23525
MSE		0.00080	0.00058	0.00039	0.00033	0.00028	0.00026
ABI		0.01205	0.01373	0.01427	0.01435	0.01468	0.01475
BI-C		−0.10355	−0.03711	−0.00916	−0.00405	−0.00101	−0.00045
MRE		0.09293	0.07949	0.06616	0.06207	0.05972	0.05922
AVE	θ=0.35	0.32440	0.32240	0.32179	0.32172	0.32132	0.32124
MSE		0.00168	0.00137	0.00109	0.00099	0.00092	0.00089
ABI		0.02560	0.02760	0.02821	0.02828	0.02868	0.02876
BI-C		−0.04822	−0.01730	−0.00428	−0.00190	−0.00047	−0.00021
MRE		0.09734	0.08970	0.08309	0.08157	0.08200	0.08216
AVE	θ=0.50	0.44514	0.44415	0.44273	0.44242	0.44234	0.44240
MSE		0.00438	0.00396	0.00371	0.00360	0.00346	0.00341
ABI		0.05486	0.05585	0.05727	0.05758	0.05766	0.05760
BI-C		−0.02734	−0.00978	−0.00244	−0.00108	−0.00027	−0.00012
MRE		0.11523	0.11327	0.11468	0.11519	0.11532	0.11520
AVE	θ=0.65	0.56005	0.55840	0.55781	0.55710	0.55663	0.55676
MSE		0.00963	0.00935	0.00895	0.00894	0.00887	0.00879
ABI		0.08995	0.09160	0.09219	0.09290	0.09337	0.09324
BI-C		−0.02390	−0.00854	−0.00212	−0.00094	−0.00023	−0.00010
MRE		0.13896	0.14096	0.14182	0.14292	0.14365	0.14345
AVE	θ=0.83	0.69453	0.69328	0.69296	0.69238	0.69203	0.69217
MSE		0.01961	0.01948	0.01925	0.01919	0.01916	0.01908
ABI		0.13547	0.13672	0.13704	0.13762	0.13797	0.13783
BI-C		−0.03173	−0.01124	−0.00278	−0.00123	−0.00031	−0.00014
MRE		0.16322	0.16472	0.16511	0.16580	0.16623	0.16606
AVE	θ=0.95	0.80194	0.80115	0.80106	0.80066	0.80044	0.80056
MSE		0.02268	0.02263	0.02241	0.02246	0.02244	0.02238
ABI		0.14806	0.14885	0.14894	0.14934	0.14956	0.14944
BI-C		−0.06013	−0.02111	−0.00519	−0.00229	−0.00057	−0.00025
MRE		0.15585	0.15668	0.15678	0.15720	0.15743	0.15730

**Table 4:** Some descriptive statistics for remission times, numbers of fires, and COVID-19 data.

Data	Min	1st Qu.	Median	Mean	3rd Qu	Max
Data Set I	1.00	7.00	16.50	19.55	28.25	49.00
Data Set II	0.00	2.00	4.00	5.40	8.00	43.00
Data Set II	1.00	4.00	7.00	8.34	12.50	22.00

**Table 5:** Fitted estimates for remission times, numbers of fires, and COVID-19 data.

Data	Model	Estimates		KS	*p*-Value
Data Set I	NDL(θ)	0.089342(0.013524)		0.11756	0.94505
	DL(θ)	0.095408(0.015115)		0.12546	0.91128
	Gc(θ)	0.048662(0.010609)		0.14475	0.79613
	DB(α,θ)	18.627559(38.92987)	0.979964(0.041469)	0.34111	0.01904
	DP(θ)	0.695781(0.056437)		0.35630	0.01247
	DBH(λ)	0.998365(0.009288)		0.751305	0.00000
Data Set II	NDL(θ)	0.250054(0.014091)		0.13702	0.01974
	DL(θ)	0.300157(0.019435)		0.15155	0.00703
	Gc(θ)	0.156290(0.012944)		0.16364	0.00276
	DB(α,θ)	2.502556(0.486995)	0.761172(0.042739)	0.19247	0.00022
	DP(θ)	0.546251(0.029829)		0.249597	0.00000
	DBH(λ)	0.983652(0.012697)		0.54740	0.00000
Data Set III	NDL(θ)	0.181266(0.017119)		0.09331	0.80782
	DL(θ)	0.206932(0.021521)		0.10142	0.71910
	DB(α,θ)	32.10536(34.10685)	0.983601(0.017297)	0.29787	0.00048
	DP(θ)	0.617625(0.043428)		0.30551	0.00031
	Gc(θ)	0.107062(0.014756)		0.21650	0.02441
	DBH(λ)	0.991326(0.014370)		0.67242	0.00000
